# Using MRI to evaluate and predict therapeutic success from depot-based cancer vaccines

**DOI:** 10.1038/mtm.2015.48

**Published:** 2015-12-16

**Authors:** Drew R DeBay, Kimberly D Brewer, Sarah A LeBlanc, Genevieve M Weir, Marianne M Stanford, Marc Mansour, Chris V Bowen

**Affiliations:** 1 Biomedical Translational Imaging Centre (BIOTIC), Halifax, Nova Scotia, Canada; 2 Immunovaccine Inc., Halifax, Nova Scotia, Canada; 3 Department of Radiology, Dalhousie University, Halifax, Nova Scotia, Canada

## Abstract

In the preclinical development of immunotherapy candidates, understanding the mechanism of action and determining biomarkers that accurately characterize the induced host immune responses is critical to improving their clinical interpretation. Magnetic resonance imaging (MRI) was used to evaluate *in vivo* changes in lymph node size in response to a peptide-based cancer vaccine therapy, formulated using DepoVax (DPX). DPX is a novel adjuvant lipid-in-oil–based formulation that facilitates enhanced immune responses by retaining antigens at the injection site for extended latencies, promoting increased potentiation of immune cells. C57BL/6 mice were implanted with C3 (HPV) tumor cells and received either DPX or control treatments, 5 days post-implantation. Complete tumor eradication occurred in DPX-vaccinated animals and large volumetric increases were observed in the vaccine-draining right inguinal lymph node (V_RILN_) in DPX mice, likely corresponding to increased localized immune response to the vaccine. Upon evaluating the relative measure of vaccine-potentiated immune activation to tumor-induced immune response (V_RILN_/V_LILN_), receiver-operating characteristic (ROC) curves revealed an area under the curve (AUC) of 0.90 (±0.07), indicating high specificity and sensitivity as a predictive biomarker of vaccine efficacy. We have determined that for this tumor model, early MRI lymph node volumetric changes are predictive of depot immunotherapeutic success.

## Introduction

The clinical development of immunotherapy in cancer has been invigorated over the past few years, with several monoclonal antibodies, cytokines, two checkpoint inhibitors, and a therapeutic vaccine being approved for human use.^1–5^ The success of checkpoint inhibitors in particular has led to a renewed interest in all immunotherapies, particularly when applied in combination to boost the overall immune response. As preclinical research leads to clinical trials, understanding and quantifying the unique mechanism of action of novel immunotherapy candidates at the preclinical stage is critical. This understanding will aid in improving interpretation of clinical responses, patient stratification, dosing, and, in particular, combination of multiple therapies, which will aid in clinical trial design.

The Response Evaluation Criteria in Solid Tumors, or RECIST,^6,7^ is currently the standard metric for evaluating tumor response to cancer therapies. RECIST guidelines are based on changes in volumetric tumor burden (*i.e.*, overall tumor number and size). While RECIST has proven to be an excellent indicator of chemotherapeutic success, it is a poor biomarker for evaluating the new class of targeted therapies, including biologics, immunotherapies, and other combined therapies.^8^

Some immunotherapeutics enhance antitumor response by decreasing the metabolic activity of the tumor without immediate tumor shrinkage.^8–10^ Other immunotherapeutics can even cause a targeted immune response that results in localized tumor swelling, due in part to infiltration of tumor-targeting immune cells. This has led to the implementation of the immune-related response criteria^8^ in several clinical trials of immunotherapies, particularly trials of checkpoint inhibitors.^11,12^ While immune-related response criteria does allow for localized changes in tumor volumes due to short-lived immunotherapeutic responses, it still relies on volumetric analysis of tumors, without a more accurate representation of the underlying immune response.

It is therefore crucial to find better metrics, or biomarkers, that more accurately represent host immune responses induced by immunotherapies, particularly at early stages. These predictive biomarkers could be used to ensure that therapies are working, even if there are no immediate positive changes in tumor volume.

Recently, there has been an effort to evaluate and quantify the amount and type of immune cell infiltrating tumors and using these quantities as a predictor of host response to cancer.^13–17^ This biomarker is known as the “immunoscore” and is currently being evaluated as a predictive clinical indicator. Unfortunately, it is more invasive, requiring histopathological evaluation of tissue after surgical removal of tumor, which is problematic if the primary tumor is unavailable for resection. Additionally, it is currently used prior to immunotherapy treatment as a predictor of the state of the immune system at that time, as opposed to a direct indication of early treatment success.

There are a number of other biomarkers also being explored both at the preclinical level and in clinical trials. Some of the biomarkers of interest include expression of PD-L1 within tumors,^18^ EGFR mutations,^19^ SUMO pathway components,^20^ and genomic characteristics of tumors.^21,22^ These are all invasive, histopathology-based biomarkers, many of which require primary tumor samples for analysis, and there remains significant questions about their feasibility in larger populations. For example, the use of PD-L1 as a predictive biomarker is confounded by several issues including tissue preparation,^18^ primary versus metastatic biopsies,^23^ and intratumoral heterogeneity.^24^

While histopathological methods are certainly important, longitudinal *in vivo* evaluation of these immunotherapeutics would provide enhanced insight into their efficacy and mechanisms of action, when assessed using optimal evaluation metrics. Previously, magnetic resonance imaging (MRI) has been used to investigate the mechanism of action of a peptide-based vaccine *in vivo* by looking at the longitudinal biological clearance of individual vaccine components tagged with superparamagnetic iron oxide.^25^ For the current study, rather than looking at the vaccine, MRI was employed to evaluate changes in lymph node size that had been anecdotally observed previously.^26^ These volumetric changes may be indicative of both tumor and vaccination immune response, and our goal is to develop a biomarker that could be an early predictor of treatment success.

In this study, we used MRI to evaluate the therapeutic response induced by DepoVax (DPX), a lipid-based vaccine platform that was developed to enhance the immunological potency of peptide vaccines. The formulation results in a unique local “depot” at the site of injection that is MRI visible for several weeks.^25^ The DPX platform can be formulated with MHC I and II restricted epitope peptide antigens and an adjuvant of choice. This entraps the vaccine ingredients in a form amenable to efficient uptake and processing/presentation by antigen-presenting cells.

C3 tumor bearing mice (HPV-16 model) were used to evaluate the *in vivo* response to vaccination with DPX-based vaccines, either with or without antigen, and compared to untreated controls. MRI was used to longitudinally monitor lymph node and tumor volumes weekly over 4 weeks until study endpoint. We then evaluated changes in lymph node volumes occurring in response to therapy as potential predictive biomarkers for treatment success.

## Results

### C3 tumor challenge and vaccination assessment with MRI

Tumor establishment in most instances was near-immediate, with 16 of 21 mice having MR-visible tumors within 5–8 days of C3 cell implantation. Of the five mice without MR-visible tumors by day 5, three were from groups vaccinated with the vehicle control and two from those vaccinated with DPX. By day 23, 20 of 21 mice had at some point displayed MR-visible tumor masses, verifying overall viability of the C3 implantation technique. One DPX-vaccinated mouse did not develop a visible tumor over the course of the entire study, likely due to effective immune control of tumor cells by HPV-specific immune responses. We have previously demonstrated that control of C3 tumors requires development of an HPV-specific immune response after vaccination.^27^

**Figure 1:**
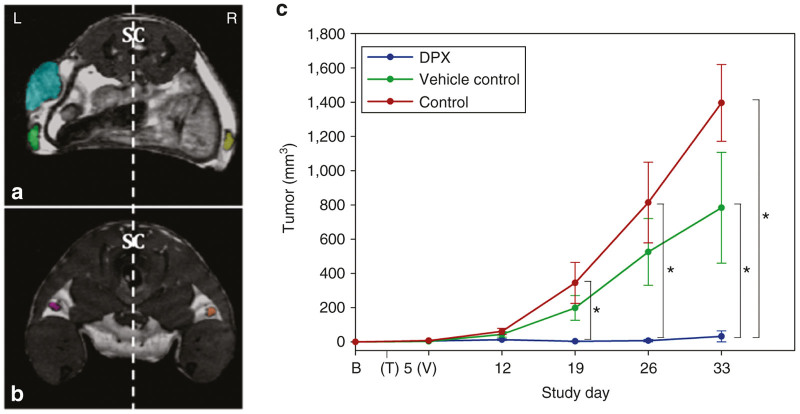
Representative MR image and tumor volumes. Axial images (150-μm isotropic voxels) showing representative segmentations of (**a**) tumor (blue), left and right inguinal LNs (green and yellow, respectively) and (**b**) left and right popliteal LNs (purple and orange, respectively). SC, spinal cord. (**c**) Mean tumor volume ± SE for each group over course of tumor challenge. * denotes statistical significance (*P* < 0.0167). Control group tumor volumes were significantly greater than DPX by days 19 and 26, and both control and vehicle control group animals bared significantly larger tumors by day 33.

**Figure 2:**
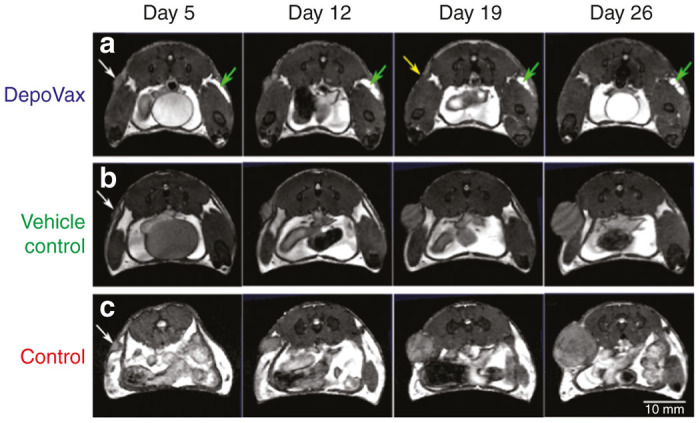
Representative MR images of tumors and vaccine sites. Representative axial images (150 μm isotropic voxels) of (**a**) DPX, (**b**) vehicle control, and (**c**) control mice over first 4 weeks of tumor challenge. White arrows indicate tumor implant sites, green arrows show depot injection sites, and yellow arrow (DPX group, day 19) indicates complete tumor eradication while evident tumor growth is seen in the non-DPX groups.

The tumors of mice in control and vehicle control groups exhibited unchecked growth over the course of the tumor challenge (Figures 1c and 2b,c), while only those mice vaccinated with DPX demonstrated successful tumor suppression. There was also complete tumor eradication in six of the seven DPX-vaccinated animals by day 26 (Figures 1c and 2a). The one DPX-vaccinated mouse that did not show complete tumor suppression appeared to be the result of vaccine injection technique, as a vaccination site was also not visible in this mouse by MRI (but was visible in all other DPX-vaccinated mice, both peptide and vehicle control). Thus, the prospect of incomplete dose administration was likely.

The marked tumor progression displayed by mice in the two control groups was significantly greater than the mice in the DPX-vaccinated group from day 19 onward and at day 33 for the vehicle control group (Figure 1c). Statistical differences in tumor size were not observed between control groups at any MRI scan interval over the course of study.

### MRI contrast and volumetry

Balanced steady-state free precession (SSFP), with the imaging parameters implemented in this study, provided sufficient signal-to-noise ratio and tumor contrast to verify the establishment of C3 tumors and delineate subsequent progression (or equivalently, eradication) over the course of the challenge. Exquisite visualization of vaccine injection sites was provided immediately upon injection, appearing as hyper-intense subcutaneous pockets on MRI, due to its lipid component (Figure 2a). Additionally, inguinal and popliteal lymph nodes (LNs) were readily distinguishable and amenable to precise segmentation, presenting on MR as dark gray structures that reside in the high-intensity fat pads of the mammary glands (Figure 1a,b). Slight chemical shift artifact, present at the sharp signal transitions between fat and LN borders, was minimal and did not impede the segmentation of these structures (Figure 1a,b).

### Monitoring systemic immune response via popliteal LNs

**Figure 3:**
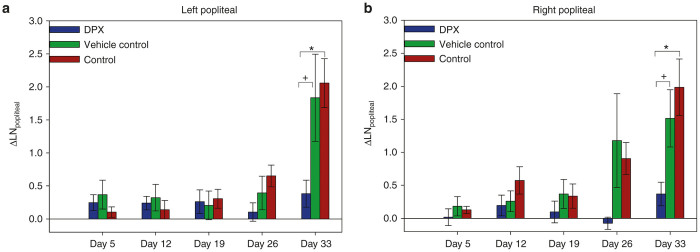
Volumetric changes in popliteal lymph nodes. Fractional volume change in (**a**) tumor-draining (left) and (**b**) vaccine draining (right) popliteal LN ±SEM. * denotes statistical significance (*P* = 0.004) and + indicates a statistical trend (*P* = 0.0214) at day 33, indicating significant enlargement of left popliteal LNs in non-DPX groups.

Distal to C3 implantation sites, both ipsilateral (left) and contralateral (right) popliteal LNs were used as a surrogate measure of systemic immune response throughout the tumor challenge. As shown for the left popliteal LN in Figure 3, the fractional volumetric change for LN of DPX-vaccinated mice remained small throughout the study (<1 relative to baseline measurements). However, the two control groups showed LN fractional volumetric increases of more than 1.5 times the baseline LN size by day 33. This represents an approximate fivefold increase in LN volume of control groups compared to the DPX-vaccinated group at this late stage of tumor challenge, where associated tumor progression was at its peak in these groups.

### Monitoring immune response at tumor site via tumor draining inguinal LN

**Figure 4:**
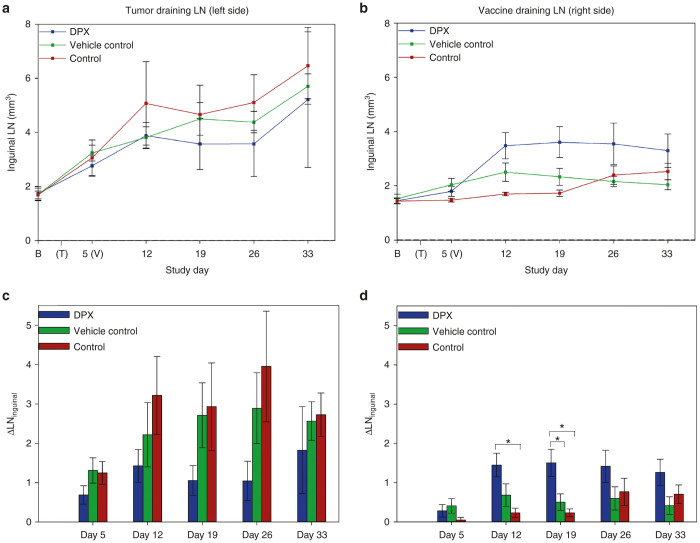
Volumetric changes in inguinal lymph nodes. **a**) Mean LN volumes (tumor side) ± SE. Repeated-measures analysis of variance (RM ANOVA) (not shown) revealed commensurate increases in left inguinal LN over tumor challenge for both control and vehicle control groups, though no intergroup differences were seen at any time point. (**b**) Mean LN volumes (vaccine-draining LN) ± SE. RM ANOVA (not shown) revealed significant increases in right inguinal LNs of DPX by day 12 (after vaccination). (**c**) Fractional change in left inguinal LN ± SE. (**d**) Fractional change in right inguinal LN ± SE. * denotes statistical significance (*P* < 0.0167). Significantly greater changes in right inguinal LN volumes in DPX mice were seen by day 12 compared to the control group and to vehicle and control groups at day 19.

In order to gain insight into the associated local immune response to C3 tumors, volumetric increases from baseline in the tumor draining (left inguinal) LN were monitored over the tumor challenge. Concomitant enlargement of left inguinal LNs with tumor progression in both untreated control and vehicle control groups (over a threefold increase) was observed over the course of the challenge, as revealed by repeated-measures analysis of variance, though no intergroup differences were detected at any MRI interval (Figure 4a,c).

### Characterizing immune response to vaccine site: differential enlargement of vaccine draining inguinal LN with DPX

Vaccine draining, right inguinal LN volumes were assessed in order to characterize any vaccine-induced immune response by the different vaccine formulations. A significant, twofold enlargement of right inguinal LNs in DPX-vaccinated animals was seen immediately at day 12 (within 1 week of vaccination), and these values remained elevated over the course of the study (Figure 4b). This corresponds to peak ELISPOT immune responses typically detected between days 7 and 10 post vaccination.^27,28^ The LNs of mice in the vehicle control group, containing adjuvant alone, failed to exhibit a similar response. In the period of activity coinciding with tumor eradication (day 19), DPX draining LNs were significantly greater than both non-DPX control counterparts (Figure 4d).

We hypothesize that the size ratio between the vaccine draining inguinal LN and tumor draining inguinal LN may be an informative biomarker, predicting efficacy of immunotherapy. This can be assessed in this particular study design by computing the volumetric ratio of the vaccine draining (right) inguinal LN (V_RILN_) relative to the tumor draining (left) inguinal LN (V_LILN_). In what we will refer to as the immune activity index V_RILN_/V_LILN_ provides a correlate of immune activity induced in the vaccine draining relative to tumor-draining lymphatic system response in an animal at any given time point. Asymmetric bilateral LN enlargement would therefore indicate a highly vaccine favored *in vivo* response (V_RILN_/V_LILN_ > 1) or alternatively a highly tumor-induced response (V_RILN_/V_LILN_ < 1).

**Figure 5:**
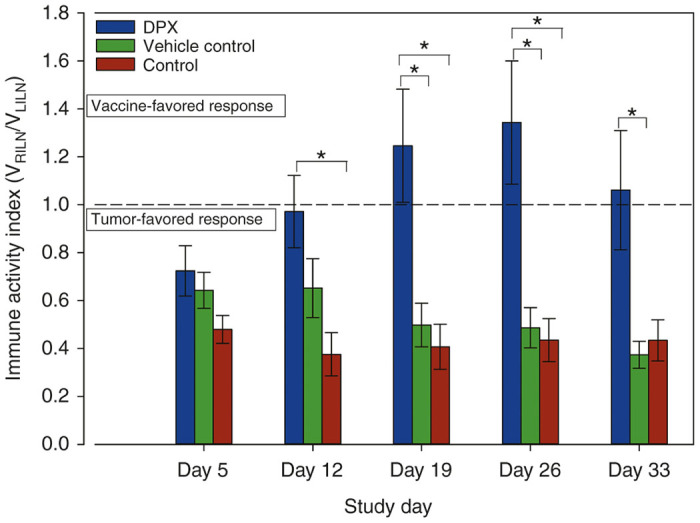
Immune activity index indicating the proportion of immune response attributed to either tumor implant site or vaccine injection site. Data presented as group means ± SEM over the period of study. * denotes statistical significance, where *P* ≤ 0.0032.

Calculation of this immune activity index revealed asymmetric left LN enlargement at day 5 of the study in all groups in response to C3 cell implantation (Figure 5). By day 12 (1 week after vaccination), a clear shift toward a vaccine-sided response was evident in DPX-receiving mice alone, and in days 19–33, this index was significantly greater than for both groups of control mice.

### Assessing diagnostic accuracy of vaccine draining LN enlargement and associated metrics: a prognostic biomarker of vaccine efficacy?

**Figure 6:**
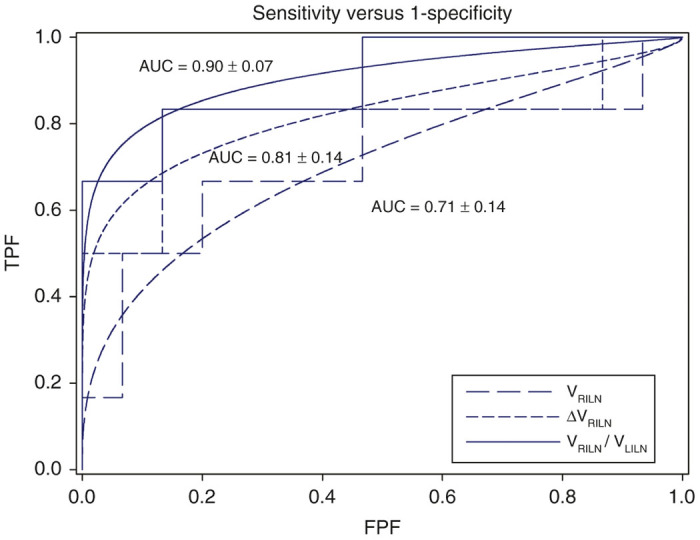
Receiver-operating characteristic curves illustrating sensitivity (TPF) versus 1-specificity (FPF) for vaccine draining LN metrics, immune activity index (V_RILN_/V_LILN_), fractional change in V_RILN_ (ΔV_RILN_), and V_RILN_ at day 19. With an AUC of 0.9 ± 0.07 (95% confidence interval = 1.0/0.74), V_RILN_/V_LILN_ provides positive predictive value for successful immunotherapeutic eradication of tumors.

In order to evaluate the prospect of using this volumetric characterization of immune response (right LN enlargement) as a prognostic predictor of vaccine efficacy accompanying tumor eradication, LN volumetry and associated metrics were assessed using receiver-operating characteristic (ROC) curves. Success outcome criteria were defined as complete tumor eradication on day 33 MRI images. True-positive and false-positive classifications were assigned to all 21 animal’s vaccine draining LN metric (either V_RILN_/V_LILN_, V_RILN_, or ΔV_RILN_) and observed (tumor/no tumor) outcome measure. The immune activity index, V_RILN_/V_LILN_, provided an area under the ROC curve (AUC) of 0.90 ± 0.07, with an upper/lower 95% confidence interval of 1.0/0.74 (Figure 6). RILN volume (V_RILN_) or ΔV_RILN_ alone performed less well with lower AUCs (0.71 ± 0.14 and 0.81 ± 0.07, respectively) and much larger 95% confidence intervals (1.0/0.40 and 1.0/0.53 (Figure 6), suggesting the immune activity index metric may provide enhanced diagnostic performance compared to vaccine-draining LN metrics alone.

## Discussion

### Balanced SSFP MRI imaging

Balanced SSFP has a proven track record for providing high signal-to-noise ratio, good fat/water contrast in various applications and established use in preclinical oncological studies, including those involving lymph node imaging.^29^ In this study, it has also proven to be a robust imaging sequence for tumor challenge applications of this nature involving the assessment of depot vaccine formulations.

Despite the presence of adjuvant in vaccine delivered to the vehicle control animals, statistical differences in tumor size were not observed between the two control groups at any MRI scan interval over the course of the study. This result indicates that tumor suppression and eradication provided by the DPX formulation is not attributed to adjuvant delivery alone, but rather requires effective, sustained delivery of both adjuvant and antigen.^25^ Direct *in vivo* visualization of DPX persistence over this longitudinal study (Figure 2, green) also corroborates earlier evidence of biodistribution, suggesting a time scale of weeks to months for depot clearance.^25^

### Late-stage, tumor-induced systemic immune activity

The drastic bilateral popliteal LN enlargement seen at study day 33 in the non–DPX-treated groups suggests a generalized state of hyperimmune activity induced by aggressive tumor growth in the late stages of tumor progression. LN swelling in response to large tumors is commonly observed in human cancers and may be the result of increased antigenicity of large tumors stimulating the immune system; however, as evidenced by the continued growth of the tumor, this response is ineffective at suppressing tumor growth.^30^ Advanced tumors can produce several mediators of inflammation to induce immune suppression, which can result in regional LN swelling.^31^

### Immune system response at tumor site

The tumor-draining inguinal LN enlargement (over a threefold increase in both control groups beyond day 19) is suggestive of a mounting immune response to C3 cell implantation (similar results were seen in a 4T1 mouse model^32^); however, the capacity of DPX to modulate this response remains unclear as intergroup comparisons also proved statistically inconclusive (however, repeated-measures analysis of variance indicates that DPX tumor draining LN alone did not increase over study). Volumetric assessment alone obviously cannot fully explain events at the cellular level; however, a physiological interpretation of this result could be that in the case of DPX-vaccinated mice, tumors are being eradicated, therefore, less aggressive sequestration of T lymphocytes is required which manifests itself as less drastic tumor draining LN enlargement.

### Characterizing immune response to vaccine site

The differential enlargement of vaccine draining LNs initiated by day 12 and seen only in DPX receiving animals is highly suggestive of increased clonal T-cell production in this proximal inguinal LN. Notably, we typically detect peak vaccine-induced immune responses using ELISPOT (data not shown) between 7 and 10 days after immunization,^27,28^ which corresponds to study day 12. These results are in alignment with a recent study^33^ that demonstrated increasing DC migration to the vaccine site correlates with improving anti-tumor immunotherapy. Though perhaps not surprising, this direct assessment provides important insight into the temporal dynamics of DPX’s immune activation profile, *in vivo*, that until now has not been completely characterized or understood. The efficacy of a vaccine is dependent on its priming immunogenicity and capacity to maintain the immune response through sustained delivery of antigen and adjuvant.^34^ A vaccine’s ability to modulate an efficacious immune response relies on both its ability to prime the immune response and then to maintain stimulation by providing sustained delivery of antigen and adjuvant over a prolonged period. This volumetric characterization of vaccine draining LN enlargement provides *in vivo* evidence of a sustained potentiation of immunotherapy response by a depot vaccine. The differential and sustained immune response potentiated by DPX is unique to this formulation and may be an important factor that contributes to the observed efficacy of DPX.

It has been shown previously that LN size in 4–6-week-old C57BL/6 mice remain constant at this level of maturity^35^ and so the volumetric changes observed in this study can therefore be attributed primarily to immune response and not be confounded by other factors related to growth and aging. Baseline LN volumes acquired in naive mice prior to tumor challenge agree well with what has previously been reported in the literature for the strain and age of mice used in this study.^35^ It is also well established that LN enlargement alone does not provide a complete picture of immune response in the lymphatic system. Although not explicitly addressed in the current study, LN morphology and shape can be indicators of immune activity as well as underlying pathology and is worthy of consideration in future analyses.

Asymmetric bilateral LN enlargement in the DPX vaccinated group indicated a highly vaccine-favored *in vivo* response (V_RILN_/V_LILN_ > 1) upon vaccination that was not present in either of the control groups, both of which favored a tumor-induced (V_RILN_/V_LILN_ < 1) response in the study. It is crucial that potential treatments have a directed, active immune response such as the one demonstrated here by the DPX group, as opposed to a general immune stimulation (*e.g.*, the vehicle control) in order to properly eliminate the tumors. This immune activity index is of particular value in that it provides an instantaneous view of how the vaccine is performing in a tumor challenge compared to the ineffective immune response modulated by tumor implantation. Additionally, even though this metric looks at the vaccine and tumor draining LNs, there is evidence of systemic responses due to the clearance of distal tumors. In previous work, immunological responses were detected via ELISPOT from spleen cells,^28^ providing further evidence of a systemic, but tumor-directed response. The immune activity index metric can be acquired at any point in a tumor challenge (ideally 2–3 weeks after vaccination) and does not require baseline anatomical/volumetric assessment scans, or time-course information from multiple scans over a longitudinal study.

ROC curves were employed in an attempt to determine whether or not vaccine draining LN enlargement (and associated metrics) can discriminate between successful or unsuccessful vaccine-mediated tumor eradication.

We have shown that the immune activity index offers positive predictive value (before complete tumor eradication has occurred in many cases), providing both a diagnostically accurate and useful vaccine performance measure that can act as an effective biomarker to indicate successful therapeutic outcome. Although the IFN-γ ELISPOT assay is often used as an indication of immune response in preclinical and clinical studies, the responses do not always predict treatment outcome.^36–38^

### Study limitations and future work

Despite the inherent variability in tumor growth and associated immune response (as apparent by the considerable magnitude of the standard errors in measurements), at a reasonably low sample size “*n*”, we were able to detect significant changes in LN volumes that can greatly assist the characterization of vaccine-induced immune responses. However, it would be very reasonable to acknowledge that this study may be statistically underpowered to detect any effect in the observed immune response to tumor implantation at the left inguinal LN level where competing processes may make any distinctions more difficult to detect at relative low sample size. The study design involves specific knowledge of primary tumor and vaccine injection sites that drain normally symmetric bi-lateral LNs; however, primary tumor location is often known in preclinical (or clinical) settings, and the vaccine injection site can be altered to accommodate such image-based biomarkers. This approach offers promise for clinically translatable imaging biomarkers to assess immunotherapeutic response.

### Conclusions

This proposed indication of MRI assessment of the therapeutic efficacy of depot vaccine formulations proves to be one of great promise. The inherent contrast due to the composition of depot vaccines in general makes its visualization by imaging readily achievable. This will help in further elucidating the mechanism of action of these depot formulations and help assess vaccine clearance behavior and the role that each vaccine component plays in effective therapies. More importantly, we have determined that MRI is capable of detecting early volumetric changes in LNs *in vivo* that may be predictive of early therapeutic success. This imaging biomarker could allow for more effective preclinical evaluation of depot vaccine formulations and also has strong potential for clinical use, particularly to improve translation of these therapies from the bench to the patient.

## Materials and Methods

### Mice

C57BL/6 female mice (4–6 weeks old, pathogen free) were obtained from Charles River Laboratories (Wilmington, MA) and housed with food and water *ad libitum* under filter top conditions. Experiments involving the use of mice were carried out in accordance with protocols approved by the University Committee on Laboratory Animals at Dalhousie University, Halifax, Nova Scotia, Canada.

### C3 cell line

HPV-16 E7-expressing C3 tumor cells were used as described previously.^28,39^ Briefly, cells were maintained in Iscove Modified Dulbecco’s medium (Sigma, St Louis, MO) and supplemented with 10% heat-inactivated fetal calf serum (Sigma), 2 mmol/l l-glutamine (Gibco, Burlington, Ontario, Canada), 100 U/ml penicillin, and 100 μg/ml streptomycin (Gibco). Cells were incubated at 37 °C/5% CO_2_. C3 cells were grown to 95% confluency and harvested with 0.05% trypsin prior to tumor challenge implantation.

### Peptide antigen and adjuvant

The HPV-16 E7 (H-2D^b^) peptide RAHYNIVTF49–57 (R9F)^39^ was fused to the universal CD4^+^ T-helper epitope PADRE^40^ (Dalton Chemical Laboratories, Toronto, Ontario, Canada). This peptide was used in vaccines at 50 μg/dose and is henceforth termed FP. Peptide vaccines were formulated in DepoVax as previously described.^28,41^

### Vaccine formulations

Vaccine formulations were administered in three groups, namely, (i) DepoVax (DPX) containing FP and adjuvant, (ii) a vehicle control vaccine, comprised DPX-containing adjuvant and no FP, and (iii) an unvaccinated control group. Vaccine liposomes were prepared following methods described previously.^28^ DepoVax is a proprietary vaccine formulation to Immunovaccine (Halifax, Canada).

### C3 tumor challenge and immunization

Mice were implanted with 5 × 10^5^ C3 tumor cells s.c. in the left flank at day 0. Five days post-implantation, mice received (i) DPX (*n* = 7), (ii) peptide-free vehicle control vaccine (*n* = 7), or (iii) no injection (*n* = 7) serving as an unvaccinated control. Vaccine formulations were delivered via single 50 μl contralateral immunization (s.c., right flank). Tumor volumes were approximated weekly via caliper measurements using the following ellipsoid tumor estimate formula: long axis × (short axis)^2^/2. Tumors were classified as nonpalpable, palpable (nonmeasureable), or approximated by the aforementioned formula. Any animals with tumors exceeding 2,000 mm^3^, or with signs of ulcerations at tumor site, were immediately sacrificed. Animals were imaged with MRI over the course of a 5-week tumor challenge.

### Magnetic resonance imaging

All MRI scans were performed at 3.0 T using a Magnex Scientific clinical MR “head only” scanner (Oxford, UK) retrofitted for small animal imaging (Magnex Scientific gradient coil, ID of 21 cm; maximum gradient strength of 200 mT/m) and interfaced with a Direct Drive spectrometer (Varian, Palo Alto, CA). A 25 mm ID “Litzcage” quadrature radiofrequency coil (Doty Scientific, Columbia, SC), tuned to 128.8 MHz, was used as a transmit/receive volume coil for imaging. *In vivo* anatomical images were obtained using a 3D balanced SSFP imaging sequence (T_2_/T_1_ weighting), chosen due to the high signal-to-noise ratio and inherent fat/water contrast demonstrated by the sequence.^42^ Repetition time (T_R_), echo time (T_E_), flip angle, and bandwidth were optimized for best overall image quality in the key areas of interest which included tumor and vaccine sites and neighboring lymph nodes. The sequence consisted of T_R_/T_E_ = 8/4 ms, flip angle = 30°, and bandwidth = 50 kHz. A field of view of 38.4 × 25.5 × 25.5 mm with matrix dimensions 256 × 170 × 170 was used to acquire (150 μm)^3^ isotropic resolution images with six signal averages. Two radiofrequency phase cycled acquisitions were acquired with maximum intensity projection post-processing to suppress banding artifact (~48 minutes per MRI scan). This field of view permitted simultaneous imaging of tumor implantation site, vaccine injection site, as well as inguinal and popliteal LN, the main tumor/vaccine draining and distal LNs, respectively.

Mice were anesthetized with isofluorane, and respiration and temperature were monitored using an MRI-compatible physiological monitoring and gating system (SA Instruments, Stony Brook, NY).

Baseline anatomical MRI scans were performed 1 week prior to tumor implantation to determine pre-challenge LN volumes. Mice were subsequently imaged weekly starting on days 5, 12, 19, 26, and 33 after tumor implantation, for a total of six longitudinal time points in the tumor challenge.

### MRI image analysis

Volumetric segmentation of structures was performed by a single observer in a blinded manner to eliminate the prospect of observer bias.

All images were first zero-padded (interpolated to higher resolution grid to increase the effective resolution and image quality) using ImageJ (NIH). Images were co-registered in RView for each mouse.^43,44^ A semi-automated region growing algorithm was implemented to perform individual 3D segmentations to determine (i) C3 tumor volumes, (ii) left inguinal, (iii) right inguinal, (iv) left popliteal, and (v) right popliteal lymph node volumes (Figure 1a,b).

### Statistical analysis

Statistical comparisons of each of the aforementioned volumetric variables measured (except where noted) were made via intergroup analysis of variance of group means (grouped by day of tumor challenge), followed by Bonferroni–Dunn *post hoc* tests, where significant differences were concluded at a significance level of 1.67% (*P* < 0.0167 with Bonferroni correction after multiple (three) comparisons). Statistical trends were concluded at *P* < 0.0334 (Bonferroni corrected). To examine within-group effects seen longitudinally, two-way repeated-measures analysis of variance was performed. All data are presented as group means ± SEM.

### ROC analysis

LN volumetry and associated metrics were assessed using ROC curves.^45^ Tumor eradication was deemed successful if the final day 33 MR images revealed no evidence whatsoever of tumor mass present. The false-positive rate was calculated as FP/(FP+TN), and the true-positive rate was calculated as TP/(TP+FN) at each week of the study and were compared in ROC space using “ROC-KIT” ROC analysis software.^46–49^ ROC curves (sensitivity versus 1-specificity) were generated at day 19 of the study using a number of potential imaging biomarkers described in the Results. The AUC is a common summary measure of a diagnostic test’s performance, interpreted as the average sensitivity for all possible values of specificity.^45^ AUC represents the overall performance and diagnostic accuracy of a test, with values approaching 1 indicating perfect accuracy. AUC was measured from the empirical curve and not the fitted data in order to avoid incorrect assumptions as to the parametric distribution of the data.^45,46^
